# Epigenetic Dominance of Prion Conformers

**DOI:** 10.1371/journal.ppat.1003692

**Published:** 2013-10-31

**Authors:** Eri Saijo, Hae-Eun Kang, Jifeng Bian, Kristi G. Bowling, Shawn Browning, Sehun Kim, Nora Hunter, Glenn C. Telling

**Affiliations:** 1 Prion Research Center (PRC) and Department of Microbiology, Immunology and Pathology, Colorado State University, Fort Collins, Colorado, United States of America; 2 Department of Microbiology, Immunology and Molecular Genetics, University of Kentucky, Lexington, Kentucky, United States of America; 3 The Roslin Institute and the University of Edinburgh, Midlothian, United Kingdom, and Cellular and Molecular Biology (CMB) Program and Molecular and Cellular Integrated Neuroscience (MCIN) Program, Colorado State University, Fort Collins, Colorado, United States of America; University of Alberta, Canada

## Abstract

Although they share certain biological properties with nucleic acid based infectious agents, prions, the causative agents of invariably fatal, transmissible neurodegenerative disorders such as bovine spongiform encephalopathy, sheep scrapie, and human Creutzfeldt Jakob disease, propagate by conformational templating of host encoded proteins. Once thought to be unique to these diseases, this mechanism is now recognized as a ubiquitous means of information transfer in biological systems, including other protein misfolding disorders such as those causing Alzheimer's and Parkinson's diseases. To address the poorly understood mechanism by which host prion protein (PrP) primary structures interact with distinct prion conformations to influence pathogenesis, we produced transgenic (Tg) mice expressing different sheep scrapie susceptibility alleles, varying only at a single amino acid at PrP residue 136. Tg mice expressing ovine PrP with alanine (A) at (OvPrP-A136) infected with SSBP/1 scrapie prions propagated a relatively stable (S) prion conformation, which accumulated as punctate aggregates in the brain, and produced prolonged incubation times. In contrast, Tg mice expressing OvPrP with valine (V) at 136 (OvPrP-V136) infected with the same prions developed disease rapidly, and the converted prion was comprised of an unstable (U), diffusely distributed conformer. Infected Tg mice co-expressing both alleles manifested properties consistent with the U conformer, suggesting a dominant effect resulting from exclusive conversion of OvPrP-V136 but not OvPrP-A136. Surprisingly, however, studies with monoclonal antibody (mAb) PRC5, which discriminates OvPrP-A136 from OvPrP-V136, revealed substantial conversion of OvPrP-A136. Moreover, the resulting OvPrP-A136 prion acquired the characteristics of the U conformer. These results, substantiated by in vitro analyses, indicated that co-expression of OvPrP-V136 altered the conversion potential of OvPrP-A136 from the S to the otherwise unfavorable U conformer. This epigenetic mechanism thus expands the range of selectable conformations that can be adopted by PrP, and therefore the variety of options for strain propagation.

## Author Summary

Prions are infectious proteins, originally discovered as the cause of a group of transmissible, fatal mammalian neurodegenerative diseases. Propagation results from conversion of the host-encoded cellular form of the prion protein to a self-propagating disease-associated conformation. It is believed that the self-propagating pathogenic form exists in a variety of subtly different conformations that encipher prion strain information. Here we explored the mechanism by which prion protein primary structural variants, differing at only a single amino acid residue, interact with prion strain conformations to control disease phenotype. We show that under conditions of co-expression, a susceptible prion protein variant influences the ability of an otherwise resistant variant to propagate an otherwise unfavorable prion strain. While this phenomenon is analogous to the expression of genetically-determined phenotypes, our results support a mechanism whereby dominant and recessive prion traits are epigenetically controlled by means of protein-mediated conformational templating.

## Introduction

Prion-mediated phenotypes and diseases result from the conformationally protean characteristics of particular amyloidogenic proteins. The prion state has the property of interacting with proteins in their non-prion conformation, thus inducing further prion conversion. The prion phenomenon has been described for a variety of different proteins involved in diverse biological processes ranging from translation termination in yeast, memory in *Aplysia*, antiviral innate immune responses [1], and most recently the action of the p53 tumor suppressor [2]. Since the prion and non-prion conformations have differing biological properties, the net result of this replicative process is protein-mediated information transfer, the characteristics of which vary from prion to prion. The ubiquity of prion replication indicates that this is a wide-ranging of means of information transfer in biological systems.

In the case of mammalian neurodegenerative diseases the prion state is pathogenic as well as transmissible. A hallmark of such conditions is the inexorable progression of pathology between synaptically connected regions of the central nervous system (CNS), consistent with advancing cell-to-cell prion spread. Experimental transmission in several settings has been convincingly demonstrated in the case of the amyloid beta (Aβ) peptide which features prominently in Alzheimer's disease (AD), the intracytoplasmic protein tau, also involved in AD as well as various neurodegenerative diseases referred to as taopathies, and α-synuclein, the primary constituent of Lewy bodies found in Parkinson's disease (PD) [1], [3].

The prototypic and best-characterized prion diseases are the transmissible spongiform encephalopathies (TSEs) of animals and humans, including sheep scrapie, bovine spongiform encephalopathy (BSE), chronic wasting disease (CWD) of cervids, and human Creutzfeldt-Jakob disease (CJD). TSEs result from conformational conversion of the host-encoded cellular form of the prion protein, PrP^C^, to the corresponding prion, or scrapie form, PrP^Sc^. Since TSEs share numerous properties with nucleic acid-based pathogens, including agent host-range, stable strain properties, and the ability to mutate and respond to selective pressure, early researchers assumed a viral etiology for these diseases. While this is not the case, the unequivocal infectivity of TSEs set these prions apart. Their singular capacity to cause fatal neurodegeneration in genetically tractable animal models, and the ability to propagate and quantify infectivity, in vivo, in cell culture or cell-free conditions, provide unparalleled settings to elucidate general mechanisms and devise integrated therapeutic approaches for all diseases involving conformational templating [4].

TSEs have long incubation periods ranging from months to years, are invariably fatal, and currently incurable. While a variant of CJD (vCJD) is unequivocally linked to prions causing BSE [5], the zoonotic potential of other TSE's remains uncertain. Whereas all TSEs, including human genetic and sporadic forms, are experimentally transmissible, most are naturally infectious and frequently occur as unanticipated epidemics. Scrapie is one such example, and several iatrogenic epidemics have been reported. More than 1,500 sheep developed scrapie following administration of a scrapie-contaminated vaccine [6]. A similar recent event led to an ∼20-fold increase in the rate of scrapie in Italy [7].

Prion strain properties and the primary structure of PrP are the two major elements controlling prion transmission. Optimal disease progression appears to occur when the primary structures of PrP^Sc^ constituting the infectious prion, and substrate PrP^C^ expressed in the host are closely related [8]–[10]. Underscoring the importance of primary structure on transmission, susceptibility and disease presentation are strongly influenced by several *PRNP* polymorphisms in humans and animals. For example, a strong association between susceptibility/resistance to natural scrapie is associated with the valine (V)/alanine (A) dimorphism at PrP residue 136 [11]. Prion strains are classically defined by differences in incubation times, and the neuropathological profiles they induce in the CNS. Seminal studies of mink prions [12], as well as studies of human prions in Tg mice [13] indicated that strain information is enciphered within the tertiary structure of PrP^Sc^. While this remains the favored explanation for prion strain diversity, the mechanism by which primary and higher order PrP^C^ and PrP^Sc^ structures interact to influence pathogenesis are not understood.

Our previous studies demonstrated that A at ovine PrP residue 136 is a component of the monoclonal antibody (mAb) PRC5 epitope [14]. This property allowed us to use PRC5 in this study to distinguish OvPrP-A136 from OvPrP-V136, affording the opportunity to monitor allele-specific OvPrP conversion during prion infection. To accomplish this, we engineered Tg mice expressing either OvPrP-A136 or OvPrP-V136, as well as Tg mice expressing both alleles in the same neuronal populations. Here, using a combination of in vivo and in vitro approaches, we address the mechanism by which this important disease susceptibility dimorphism influences scrapie strain-specific pathogenesis.

## Results

### Transgenic mice to assess the effects of the OvPrP A/V136 dimorphism on scrapie pathogenesis

**Figure 1:**
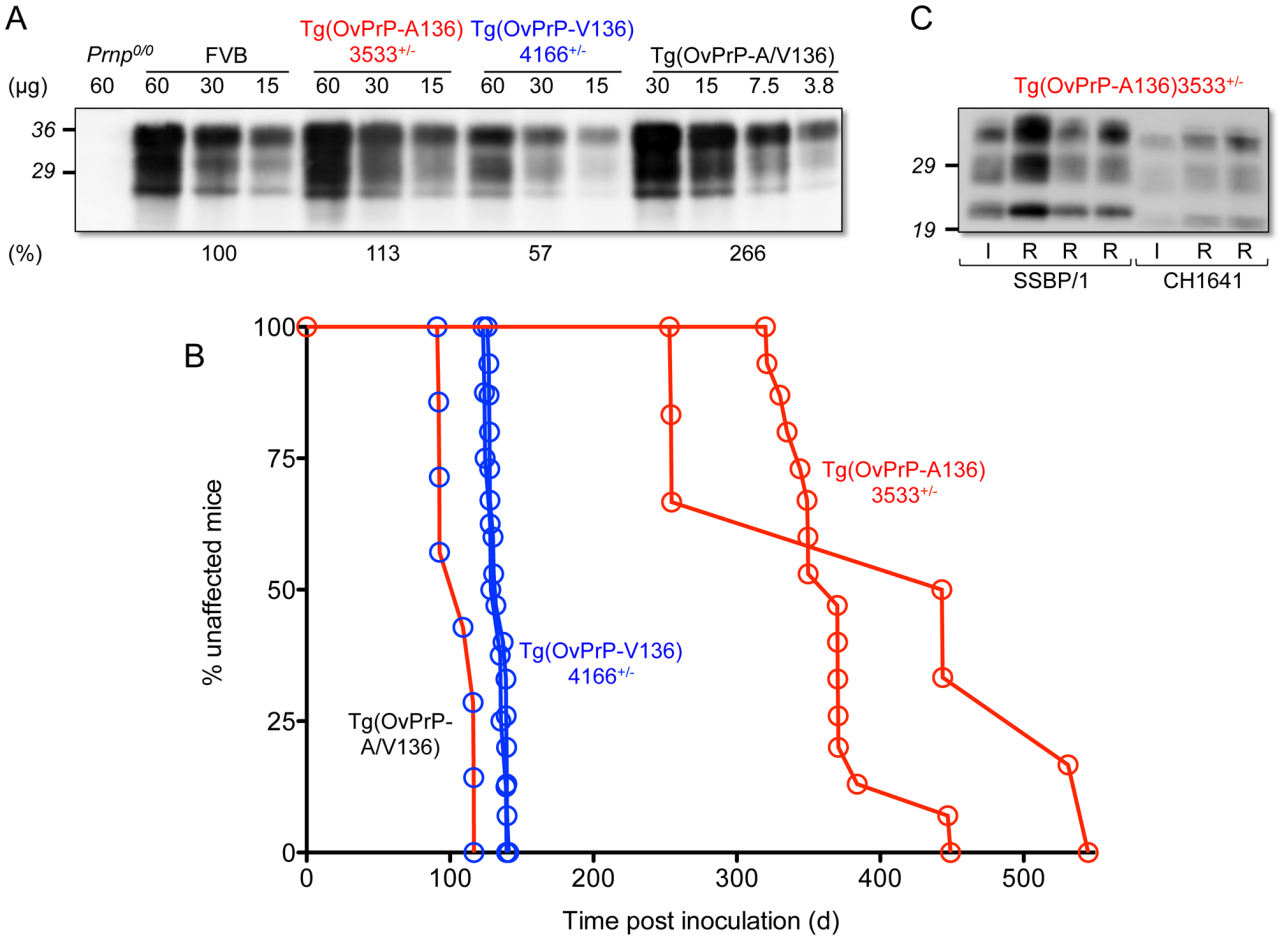
Characterization of transgenic mice expressing OvPrP-A136 and OvPrP-V136. A. Levels of transgene-expressed OvPrP in the CNS were estimated by semi-quantitative western blotting using mAb 6H4. Amounts of total protein loaded (µg) in each sample are shown. *Prnp^0/0^*, mice in which the PrP gene is disrupted; FVB, wild type mice. Estimates of expression levels, shown as a percentage (%) of that in FVB mice, are based on densitometric analysis of signals from diluted samples. **B.** Survival curves of mice following inoculation with sheep SSBP/1 scrapie prions. Percent (%) affected mice refers to numbers of mice within an inoculated cohort manifesting progressive clinical signs associated with prion disease. **C.** Western blot analysis of PK-treated brain extracts of diseased Tg(OvPrP-A136)3533^+/−^ mice. SSBP/1 and CH1641 refer to mice inoculated with the respective prions. I and R refer to sheep SSBP/1 or CH1641 inocula, and brain extracts from recipient mice respectively.

We created Tg mice expressing OvPrP encoding either A or V at residue 136. Using semi-quantitative Western and immuno dot blotting we ascertained that levels of expression in the CNS of Tg(OvPrP-A136)3533^+/−^ and Tg(OvPrP-V136)4166^+/−^ mice were close to that of PrP expressed in the CNS of wild type mice (Fig. 1A).

Both lines of Tg mice tolerated these levels of expression without spontaneously developing recognizable signs of disease (Table 1). In contrast, Tg mice of both genotypes intracerebrally (ic) inoculated with brain homogenates from scrapie-affected sheep succumbed to the neurological effects of prion disease following variable incubation periods (Table 1). Rapid disease onset occurred following inoculation of Tg(OvPrP-V136)4166^+/−^ mice with SSBP/1 prions [15], [16], which consistently produced an ∼130 d mean incubation time. While SSBP/1 also caused disease in Tg(OvPrP-A136)3533^+/−^ mice, mean incubation times were ∼230 to 280 d longer (Fig. 1B and Table 1). In contrast, CH1641 prions [17] induced disease in all inoculated Tg(OvPrP-A136)3533^+/−^ mice with a mean ∼310 d onset of disease, whereas no disease was registered in Tg(OvPrP-V136)4166^+/−^ mice after >560 d. These distinct transmission profiles are consistent with previously recognized strain differences between SSBP/1 and CH1641 scrapie prions [17]. Consistent with this notion, western blot analysis of proteinase K-treated brain extracts of diseased Tg(OvPrP-A136)3533^+/−^ mice confirmed that the molecular profiles which distinguish PrP^Sc^ constituting SSBP/1 and CH1641 prions [18] were maintained upon transmission (Fig. 1C). These results demonstrate that Tg(OvPrP-A136)3533^+/−^ and Tg(OvPrP-V136)4166^+/−^ mice are capable of distinguishing scrapie strain-specific transmission patterns, and in turn that these properties are influenced by the A/V136 dimorphism.

**Table 1:** Prion disease in transgenic mice expressing different ovine PrP scrapie susceptibility alleles.

Isolate	Genotype	Mean time to disease onset ± standard error of the mean (SEM), days^1^		
		Tg(OvPrP-V136)4166^+/−^	Tg(OvPrP-A136)3533^+/−^	Tg(OvPrP-A/V)
2 None		>552 (0/4)	>429 (0/5)	
2 SSBP/1	A136/V136	132±2 (8/8)	412±49 (6/6)	105±5 (7/7)
3 SSBP/1	A136/V136	133±1 (15/15)	367±10 (15/15)	
2 CH1641	A136/V136	>564 (0/6)	310±21 (6/6)	

1 The number of mice developing clinical signs of prion disease divided by the original number of inoculated mice is shown in parentheses.

2 Inoculations performed in Telling lab.

3 Inoculations performed in Hunter lab.

### The influence of the OvPrP A/V136 dimorphism on PrP^Sc^ conversion kinetics, conformation, and CNS deposition

**Figure 2:**
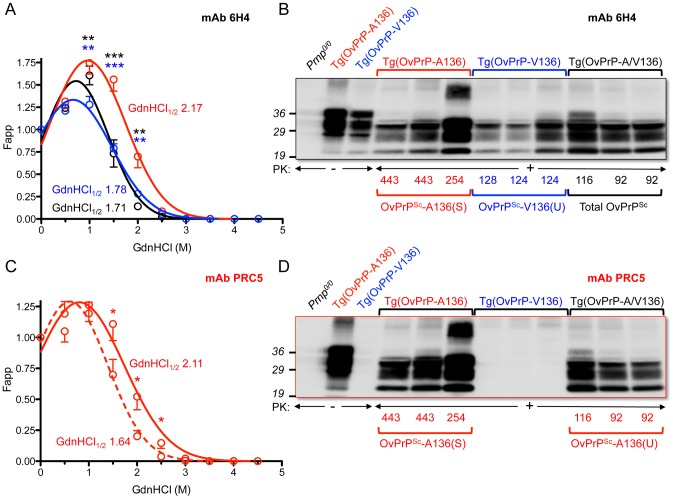
Analyses of PrP^Sc^ in the brains of SSBP/1 infected mice. In **A** and **C**, densitometric analysis of immunoblots was used to measure the amounts of protease-resistant OvPrP^Sc^ as a function of GdnHCl concentration. The dose-response curve was plotted using a Gaussian non-linear least-square fit. Each point is the mean value derived from densitometric quantification of PK-resistant PrP in three diseased mouse brains. Error bars correspond to standard errors of the mean. **A.** Conformational stability analysis using mAb 6H4 of OvPrP^Sc^-A136(S) in Tg(OvPrP-A136)3533^+/−^ mice (red line), OvPrP^Sc^-V136(U) in Tg(OvPrP-V136)4166^+/−^ mice (blue line), and total OvPrP^Sc^ in Tg(OvPrP-A/V136) mice (black line). Black asterisks compare differences between OvPrP^Sc^-A136(S) and total PrP^Sc^ in Tg(OvPrP-A136)3533^+/−^ and Tg(OvPrP-A/V) mice; blue asterisks compare differences between OvPrP^Sc^-A136(S) in Tg(OvPrP-A136)3533^+/−^ mice and OvPrP^Sc^-V136(U) in Tg(OvPrP-V136)4166^+/−^ mice. **C.** Conformational stability analysis using mAb PRC5 of OvPrP^Sc^-A136(S) in Tg(OvPrP-A136)3533^+/−^ mice (solid line) and OvPrP^Sc^-A136(U) in Tg(OvPrP-A/V136) mice (dashed line). *P<0.05, **P<0.005, ***P<0.001. In **B** and **D**, representative immunoblots of PK-resistant PrP in the brains of three mice from each infected cohort of Tg(OvPrP-A136)3533^+/−^, Tg(OvPrP-V136)4166^+/−^, and Tg(OvPrP-A/V136) mice using mAb 6H4 (**B**) and mAb PRC5 (**C**). Times of onset of disease for analyzed mice are also provided. OvPrP-V136 is indicated by blue symbols and text; OvPrP-A136 is indicated by red symbols and text.

Previous studies revealed a positive correlation between PrP^Sc^ conformational stability and the incubation times of mouse and cervid prions [19], [20], but not of hamster prions [21], [22]. We performed guanidine denaturation treatments on PrP^Sc^ in brain extracts of SSBP/1 infected Tg(OvPrP-V136)4166^+/−^ mice with short incubation times and SSBP/1 infected Tg(OvPrP-A136)3533^+/−^ mice with long incubation times. Analyses using mAb 6H4 revealed distinct stability curves for OvPrP^Sc^-V136 and OvPrP^Sc^-A136. The conformational stability of OvPrP^Sc^-V136 was lower than OvPrP^Sc^-A136 in the range of GdnHCl concentrations between 1 and 2 M, (Fig. 2A) and GdnHCl_1/2_ values were 1.78 and 2.17 respectively. This confirmed that the conformation of OvPrP^Sc^-V136 produced in Tg(OvPrP-V136)4166^+/−^ mice with rapid incubation times was less stable than OvPrP^Sc^-A136 produced in Tg(OvPrP-A136)3533^+/−^ mice with longer incubation times. We refer to these conformations as unstable (U) and stable (S), and to the rapidly and slowly propagating prions composed of these conformers as SSBP/1-V136(U), and SSBP/1-A136(S).

**Figure 3:**
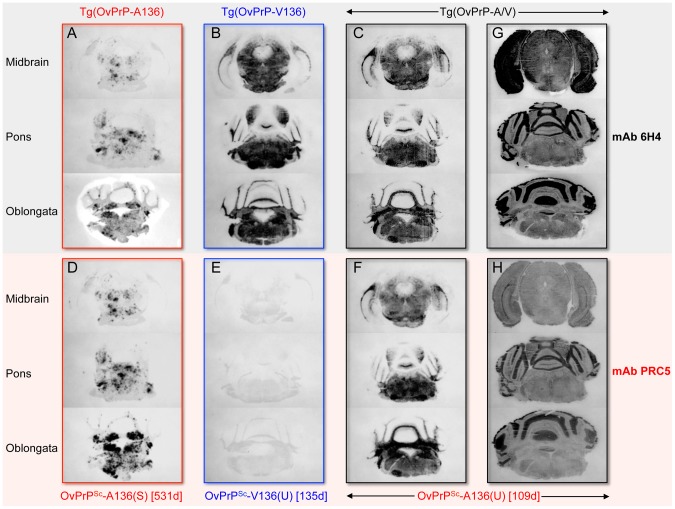
Representative OvPrP^Sc^ distribution in the CNS of diseased transgenic mice. OvPrP-V136 and OvPrP-A136 are indicated by blue and red text respectively. Times of onset of disease (d) for individual mice analyzed in histoblots are provided. Sections through the midbrain, pons and oblongata are shown for SSBP/1 infected Tg(OvPrP-A136)3533^+/−^ mice (**A** and **D**); Tg(OvPrP-V136)4166^+/−^ mice (**B** and **E**); and Tg(OvPrP-A/V) mice (**C** and **F**). In panels **G** and **H**, sections were not treated with PK and show distribution of total PrP. Histoblots in panels **A**–**G** were probed with mAb 6H4; histoblots in **D**–**H** were probed with mAb PRC5.

We then used histoblotting [23], a widely used method for characterizing strain-specific differences in PrP^Sc^ distribution [20], [24], with mAb 6H4 to characterize OvPrP^Sc^-A136(S) and OvPrP^Sc^-V136(U) deposition in the CNS. While OvPrP^Sc^-A136(S) had a punctate pattern of accumulation throughout the midbrain, pons, and oblongata of slow incubation time Tg(OvPrP-A136)3533^+/−^ mice (Fig. 3A), the neuroanatomical distribution of OvPrP^Sc^-V136(U) in the same sections of rapid incubation time Tg(OvPrP-V136)4166^+/−^ mice was distinctly different, being more intense and diffusely deposited than OvPrP^Sc^-A136(S) (Fig. 3B). Since Tg(OvPrP-A136)3533^+/−^ and Tg(OvPrP-V136)4166^+/−^ mice were both engineered using the same cosSHa.Tet cosmid vector which drives expression from the PrP gene promoter, we conclude that these differences are not the result of expression OvPrP^C^-A136 and OvPrP^C^-V136 in different neuronal populations.

### The OvPrP A/V136 dimorphism influences strain-specific PrP conversion in vitro

**Figure 4:**
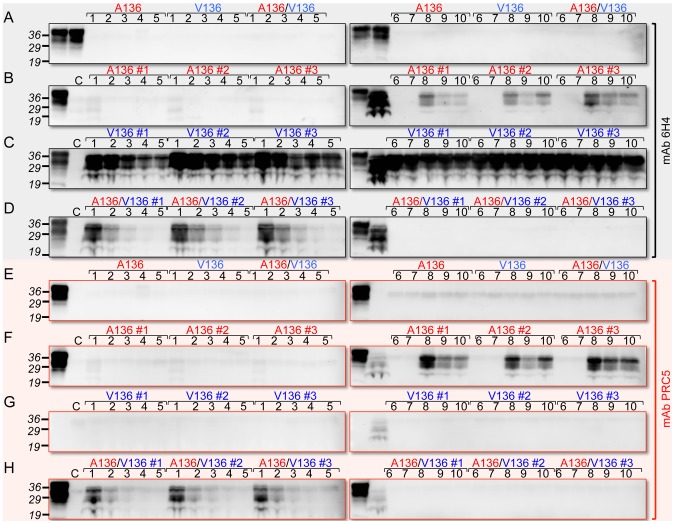
Detection of OvPrP^Sc^ by sPMCA. In **A** and **E**, the first two lanes of each panel correspond to brain extracts from Tg(OvPrP-A136)3533^+/−^ and Tg(OvPrP-V136)4166^+/−^ mice respectively without PK treatment. A136, V136, and A136/V136 refer to substrates from Tg(OvPrP-A136)3533^+/−^ mice, Tg(OvPrP-V136)4166^+/−^ mice, and mixtures of the two respectively. In **B** and **F**, the first lanes of each panel correspond to brain extracts from Tg(OvPrP-A136)3533^+/−^ mice without PK treatment; in **C** and **G**, the first lanes of each panel correspond to brain extracts from Tg(OvPrP-V136)4166^+/−^ mice without PK treatment; in **D** and **H**, the first lanes of each panel correspond to mixtures of brain extracts from Tg(OvPrP-A136)3533^+/−^ and Tg(OvPrP-V136)4166^+/−^ without PK treatment. In **B**–**D** and **F**–**H**, three different brains, indicated by #1–#3 were used as sources of substrates. In each case, the second lane of the second panel contains PK treated SSBP/1. Panels **A** and **E** are sPMCA using the indicated templates in the absence of a prion seed. Numbers in black above each lane refer to the round of sPMCA. C refers to a control in which no SSBP/1 seed was added. In each case C, and all numbered lanes were treated with PK. Blots were probed with mAbs 6H4 or PRC5 as indicated.

**Figure 5:**
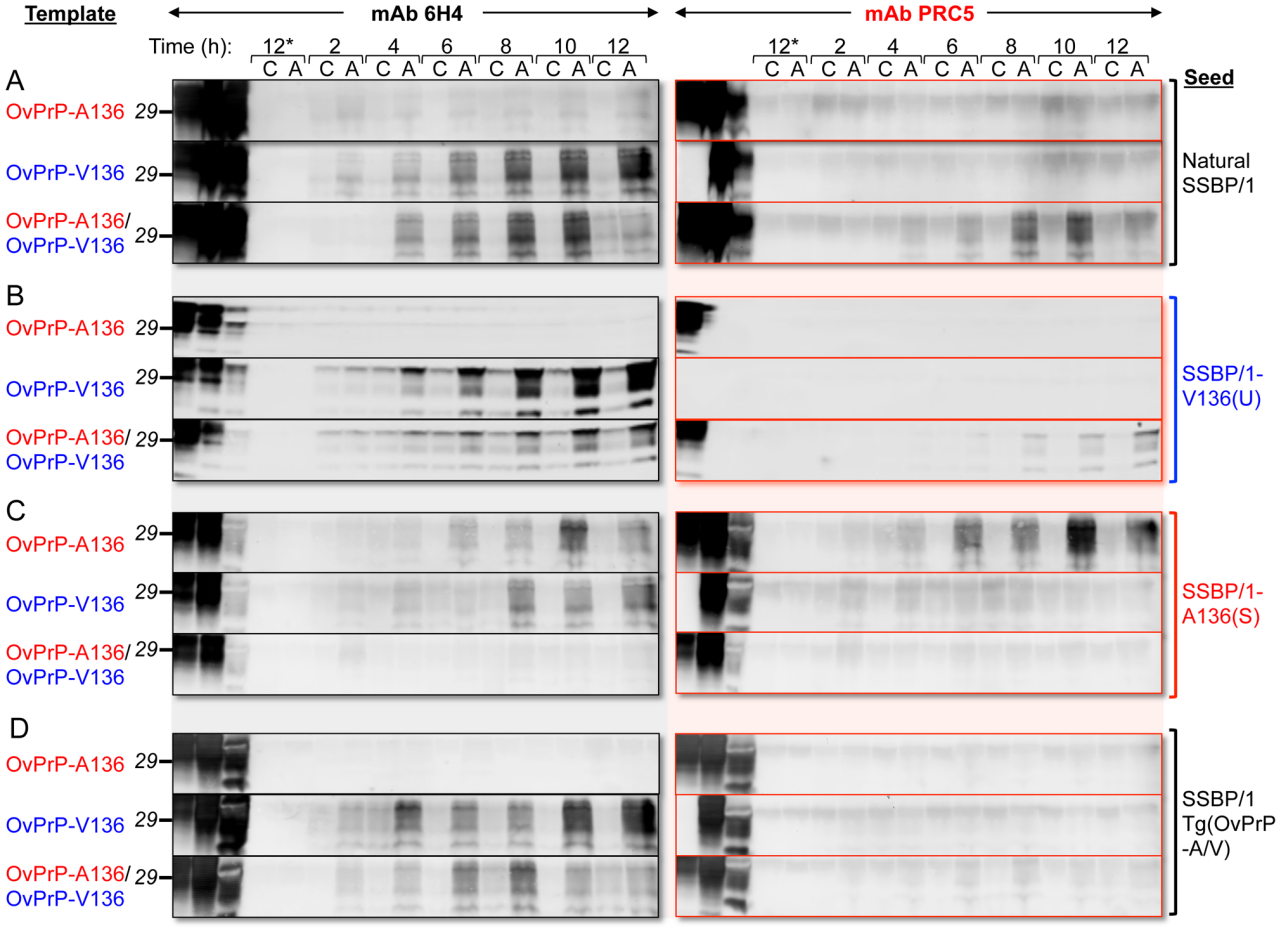
PMCA using defined seeds and substrates. PMCA was performed for various times indicated. At each time point samples were either amplified by sonication (A), or matching control samples (C) received no sonication, and were therefore not amplified. Brain homogenates from healthy Tg(OvPrP-A136)3533^+/−^ and Tg(OvPrP-V136)4166^+/−^ mice served as sources of OvPrP-A136, OvPrP-V136 or mixtures of the two. In **A**, samples were seeded with sheep SSBP/1; in **B**, samples were seeded with brain extracts from diseased Tg(OvPrP-V136)4166^+/−^ mice [SSBP/1-V136(U) prions]; in **C**, samples were seeded with brain extracts from diseased Tg(OvPrP-A136)3533^+/−^ mice [SSBP/1-A136(S); in **D**, samples were seeded with extracts from diseased Tg(OvPrP-A/V) mice. Samples labeled 12* received no seed. The first three lanes of each immunoblot were loaded with the substrate(s) for each PMCA reaction, not treated with PK; the corresponding seed, not treated with PK; and, the same seed treated with PK. All other samples were digested with PK. Western blots were probed with mAbs 6H4 and PRC5 as indicated.

We used brain extracts of Tg(OvPrP-A136)3533^+/−^ or Tg(OvPrP-V136)4166^+/−^ mice as sources of OvPrP^C^-A136 and OvPrP^C^-V136 substrates for protein misfolding amplification (PMCA) [25] using SSBP/1. While neither template spontaneously converted to PrP^Sc^ in the absence of seeded prions (Fig. 4A and E), SSBP/1 reproducibly converted OvPrP^C^-V136 to OvPrP^Sc^-V136 during a single round of PMCA (Fig. 4C). In contrast, conversion was not observed after a single round of PMCA when OvPrP^C^-A136 was used as template (Fig. 4B and Fig. 5A). We therefore used serial PMCA (sPMCA) [26] over 10 rounds to address whether conversion of OvPrP^C^-A136 to OvPrP^Sc^-A136 might be detected after prolonged replication. Serial PMCA was performed in triplicate using equal amounts of PrP from three different Tg(OvPrP-A136)3533^+/−^ or Tg(OvPrP-V136)4166^+/−^ mouse brains using sheep SSBP/1 as seed. Apart from a slight but consistent decrease between rounds two and five, OvPrP^Sc^-V136 production, detected using mAb 6H4, was sustained throughout rounds one to 10 (Fig. 4C). As expected, mAb PRC5 failed to detect OvPrP^Sc^-V136 (Fig. 4G). In contrast, OvPrP^Sc^-A136 was undetectable with mAbs 6H4 or PRC5 until round eight, after which levels decreased during rounds nine and 10 (Fig. 4B and F).

### Forced templating of a dominant PrP^Sc^ conformation in mice expressing both susceptibility alleles

Having established that Tg mice expressing OvPrP^C^-V136 and OvPrP^C^-A136 propagate SSBP/1-V136(U) and SSBP/1-A136(S) prions with relatively rapid and slow incubation times respectively, we produced Tg(OvPrP-A/V136) mice expressing both OvPrP^C^-A136 and OvPrP^C^-V136 and inoculated them with SSBP/1 to examine whether disease developed with fast, slow or intermediate kinetics. Although more rapid than the ∼130 d onset of disease in Tg(OvPrP-V136)4166^+/−^ mice (P = 0.0094), the mean 105±5 d onset of disease contrasted with the >400 d SSBP/1 incubation times observed in Tg(OvPrP-A136)3533^+/−^ mice (Fig. 1B and Table 1).

Stability assessments using mAb 6H4 showed that the denaturation curves of OvPrP^Sc^ produced in the brains of diseased Tg(OvPrP-V136)4166^+/−^ and Tg(OvPrP-A/V136) mice were superimposable over most of the range of GdnHCl concentrations (Fig. 2A), indicating that OvPrP^Sc^ produced in Tg(OvPrP-A/V) mice shared the conformation of OvPrP^Sc^-V136(U) produced in SSBP/1 infected Tg(OvPrP-V136)4166^+/−^ mice. In accordance with this notion, histoblotting using mAb 6H4 showed that the neuroanatomical distribution of OvPrP^Sc^(U) in the brains of diseased Tg(OvPrP-A/V) mice mirrored the diffuse deposition of the OvPrP^Sc^-V136(U) conformer located in similar sections of rapid incubation time Tg(OvPrP-V136)4166^+/−^ mice (Fig. 3C).

While the rapid SSBP/1 incubation times, and properties of the converted PrP^Sc^ in diseased Tg(OvPrP-A/V) were consistent with propagation of SSBP/1-V136(U) prions, remarkably, western blotting of diseased Tg(OvPrP-A/V136) brain extracts with mAb PRC5 revealed substantial conversion of OvPr^C^-A136 to OvPrP^Sc^-A136 (Fig. 2D). Densitometric comparisons of OvPrP^Sc^ levels using mAbs 6H4 (Fig. 2B) and PRC5 (Fig. 2D) allowed us to estimate relative conversion efficiencies of each allele product in the brains of SSBP/1 infected Tg(OvPrP-A/V136) mice. Using samples from diseased Tg(OvPrP-A136)3533^+/−^ mice probed with mAbs 6H4 and PRC5 as normalizing controls for differences in the affinities of the two mAbs for OvPrP-A136, we estimated by Western or dot blotting that OvPrP^Sc^-A136 comprised ∼45% of total PK-resistant PrP in the brains of diseased Tg(OvPrP-A/V136) mice.

We then used mAb PRC5 to determine the conformation of OvPrP^Sc^-A136 among total OvPrP^Sc^ produced in the brains of diseased Tg(OvPrP-A/V136) mice. The 1.64 GdnHCl_1/2_ value of OvPrP^Sc^-A136 produced under these conditions was distinct from that of OvPrP^Sc^-A136(S) produced in long incubation time Tg(OvPrP-A136)3533^+/−^ mice (GdnHCl_1/2_ = 2.11), and their non-superimposable PRC5 denaturation curves were significantly different in the range of 1.5–2.5 M GdnHCl (Fig. 2C). These findings demonstrated that the conformation of OvPrP^Sc^-A136 in rapid incubation time Tg(OvPrP-A/V) mice was distinct from OvPrP^Sc^-A136(S) produced in long incubation time Tg(OvPrP-A136)3533^+/−^ mice. We refer to this novel conformation as OvPrP^Sc^-A136(U), and to the resulting prions as SSBP/1-A136(U).

Histoblotting using mAb PRC5 confirmed the comparatively limited and punctate distribution pattern of OvPrP^Sc^-A136(S) in the CNS of long incubation time Tg(OvPrP-A136)3533^+/−^ mice (Fig. 3D) that we observed with mAb 6H4 (Fig. 3A). As expected, OvPrP^Sc^-V136(U) in the CNS of diseased Tg(OvPrP-V136)4166^+/−^ mice was refractory to detection by mAb PRC5 (Fig. 3E). We probed histoblots of the CNS from diseased Tg(OvPrP-A/V136) mice with mAb PRC5 to assess the appearance and distribution of OvPrP^Sc^-A136(U). In contrast to the punctate deposits of OvPrP^Sc^-A136(S) in long incubation time Tg(OvPrP-A136)3533^+/−^ mice (Fig. 3A and D), OvPrP^Sc^-A136(U) in Tg(OvPrP-A/V136) mice (Fig. 3F) acquired a diffuse deposition and a distribution pattern that was equivalent to OvPrP^Sc^-V136(U) in Tg(OvPrP-V136)4166^+/−^ mice (Fig. 3B). Consistent with the co-expression of each allele in identical cell populations of Tg(OvPrP-A/V) mice, spatial distributions of 6H4- and PRC5-reactive PrP coincided in all analyzed sections of Tg(OvPrP-A/V) mice (Fig. 3G and H).

### PMCA reproduces strain specific effects of one allele product on another

To simulate the combined effects of OvPrP-A136 and OvPrP-V136 on PrP conversion in Tg(OvPrP-A/V136) mice in vitro, we mixed equal quantities of OvPrP^C^-A136 and OvPrP^C^-V136 in PMCA reactions seeded with SSBP/1. Under these conditions, similar to when OvPrP^C^-V136 was present in isolation (Fig. 4C), we observed early, reproducible conversion to OvPrP^Sc^ in round one (Fig. 4D). Probing of western blots with mAb PRC5 showed that OvPrP^Sc^-A136 was a component of this converted material (Fig. 4H). Thus, similar to our observations in Tg mice, the presence of OvPrP^C^-V136 induced the relatively rapid conversion of OvPrP^C^-A136 to OvPrP^Sc^-A136 by SSBP/1. Interestingly, subsequent conversion of both OvPrP^C^-A136 and OvPrP^C^-V136 diminished in rounds two to five, ultimately becoming undetectable through rounds six to 10 (Figs. 4D and H).

SSBP/1 was originally produced from a pool of diseased sheep brains from the positive selection line in the Neuropathogenesis Unit (NPU) Cheviot sheep flock, and has subsequently been passaged as a pool. We next compared the seeding properties of SSBP/1 with those of SSBP/1-A136(S) or SSBP/1-V136(U) prions derived from SSBP/1-infected Tg(OvPrP-A136)3533^+/−^ or Tg(OvPrP-V136)4166^+/−^ mice. We monitored conversion of OvPrP^C^-A136 or OvPrP^C^-V136 templates every two hours for a total of 12 h of PMCA. SSBP/1-V136(U) had the same PMCA properties as SSBP/1: both SSBP/1 and SSBP/1-V136(U) prions efficiently converted OvPrP^C^-V136 in isolation, but not OvPrP^C^-A136 in isolation; when both templates were present in the PMCA reaction, the presence of OvPrP^C^-V136 facilitated conversion of OvPrP^C^-A136 to OvPrP^Sc^-A136 by SSBP/1 or SSBP/1-V136(U) prions (Figs. 5A and B). In contrast, SSBP/1-A136(S) prions converted either OvPrP^C^-V136 or OvPrP^C^-A136 templates to PrP^Sc^ when they were present in isolation, the latter being unequivocally confirmed to be OvPrP^Sc^-A136 using mAb PRC5 (Fig. 5C); however, in the presence of both templates SSBP/1-A136(S) prion propagation was inhibited (Fig. 5C). The properties of prions derived from Tg(OvPrP-A/V) mice differed from SSBP/1, SSBP/1-A136(S) or SSBP1/-V136(U) prions. Like SSBP/1 and SSBP/1-V136(U), prions passaged through these mice efficiently converted OvPrP^C^-V136, but not OvPrP^C^-A136. However, unlike SSBP/1 and SSBP/1-V136(U), such prions failed to facilitate conversion of OvPrP^C^-A136 in the presence of OvPrP^C^-V136 (Fig. 5D).

## Discussion

### Novel transgenic mouse models for analysis of the OvPrP-A/V136 dimorphism

Previous studies described the production of Tg mice expressing OvPrP, and reported their susceptibility to scrapie prions [27]–[32]. The most widely characterized models are tg338 mice expressing OvPrP-V136 [31], and Tgov59 [33] or Tgov4 [29] lines expressing OvPrP-A136. In the case of tg338 mice, the transgene was comprised of a bacterial artificial chromosome insert of 125 kb of sheep DNA, while in the case of Tgov59 and Tgov4 mice the neuron specific enoloase promoter was used to drive OvPrP expression. These lines are maintained on different heterogeneous genetic backgrounds, and CNS expression levels in tg338 mice are ∼8- to 10-fold higher than wild type, while Tgov59 and Tgov4 lines each over express OvPrP-ARQ at levels ∼2- to 4-fold higher than those found in sheep brain. Spontaneous neurological dysfunction has been reported in Tg lines over expressing OvPrP [27], [31].

Tg(OvPrP-A136)3533^+/−^ and Tg(OvPrP-V136)4166^+/−^ mice express transgene-encoded PrP, either slightly lower, or slightly higher than PrP levels normally expressed in the CNS of wild type mice. Since Tg(OvPrP-A136)3533^+/−^ and Tg(OvPrP-V136)4166^+/−^ lines were produced using the cosSHa.Tet cosmid vector which drives expression from the PrP gene promoter [34], we expected expression of OvPrP^C^-A136 and OvPrP^C^-V136 in identical neuronal populations, and therefore that both alleles are co-expressed in the same cells of Tg(OvPrP-A/V) mice. Finally, other than variable transgene insertion loci, both lines are otherwise sygeneic on an inbred *Prnp* ^0/0^/FVB background.

Previous studies reported on Tg mice expressing OvPrP with V at 136, referred to as Tg(OvPrP)14882^+/−^ mice, that were also produced in a *Prnp* ^0/0^/FVB background using the cosSHa.Tet cosmid vector [32]. However, in that study, comparable Tg mice expressing OvPrP-A136 were not reported. Median SSBP/1 scrapie incubation times in Tg(OvPrP)14882^+/−^mice were 75 d, and this line expresses OvPrP at levels only slightly higher than Tg(OvPrP-V136)4166^+/−^ mice. While we exercise caution when comparing results from mice produced by different groups, the otherwise similar properties of Tg(OvPrP)14882^+/−^ and Tg(OvPrP-V136)4166^+/−^ mice suggest that even slight differences in the levels of transgene expression can have significant effects on prion incubation time.

A clear link to codon 136 genotype and susceptibility/resistance to different sheep scrapie isolates has been described in multiple previous studies. Importantly, the influence of residue 136 on the transmission of SSBP/1 and CH1641 prions in Tg(OvPrP-A136)3533^+/−^ and Tg(OvPrP-V136)4166^+/−^ mice is in accordance with the properties of these isolates in sheep of various genotypes [17]. Generally, increased susceptibility to scrapie is associated with expression of OvPrP-V136, with A/A136 being the most resistant, and V/V136 the most susceptible genotypes. In the case of SSBP/1 incubation periods are ∼170 days in V/V136 sheep, while transmission to A/A136 sheep is relatively inefficient, with no disease recorded after >1000 days [35]. While SSBP/1 eventually transmits to Tg(OvPrP-A136)3533^+/−^ mice with incubation times exceeding 400 days, the general effects of the A/V136 dimorphism on SSBP/1 transmission observed in sheep are recapitulated in Tg(OvPrP-A136)3533^+/−^ and Tg(OvPrP-V136)4166^+/−^ mice (Table 1). Similarly, CH1641, which propagates efficiently in A/A136 sheep [35], preferentially propagates in Tg(OvPrP-A136)3533^+/−^ mice (Table 1). In previous studies, CH1641 transmitted to TgOvPrP4 mice with an ∼250 d mean incubation time [36].

Although SSBP/1 incubation times are prolonged in A/V136 compared to V/V 136 sheep [35], in our studies incubation times were shorter in Tg(OvPrP-A/V) than in Tg(OvPrP-V136)4166^+/−^ mice. While the condition of A/V136 heterozygosity has not been previously modeled in Tg mice, this difference may result from double the levels of transgene expression in Tg(OvPrP-A/V) mice compared to Tg(OvPrP-A136)3533^+/−^ and Tg(OvPrP-V136)4166^+/−^ mice. Tg(OvPrP-A/V136) mice were derived by mating Tg(OvPrP-A136)3533^+/+^ with Tg(OvPrP-V136)4166^+/+^ mice, and therefore express greater total levels of OvPrP than Tg(OvPrP-A136)3533^+/−^ and Tg(OvPrP-V136)4166^+/−^ mice (Fig. 1A). Since the levels of OvPrP-V136 are equivalent in Tg(OvPrP-V136)4166^+/−^ and Tg(OvPrP-A/V) mice, and we show that OvPrP-A136 also becomes available for conversion, this situation results in more available substrate for conversion. While previous studies revealed an inverse correlation between transgene expression levels and prion incubation times in Tg mice [8], whether shorter incubation periods in Tg(OvPrP-A/V136) mice than in Tg(OvPrP-V136)4166^+/−^ mice reflect overall differences in PrP^C^ expression levels remains uncertain. Differences in scrapie pathogenesis between mice and sheep may also reflect the influence of additional factors on disease in the natural host including other *PRNP* polymorphisms [37], [38], and different involvements of the lymphoreticular system in sheep compared to Tg mice.

### Novel insights into the role of the A/V136 polymorphism on strain selection and prion propagation

Our observations in Tg mice expressing individual allele products suggested that rapid or prolonged SSBP/1 incubation times in Tg(OvPrP-V136)4166^+/−^ and Tg(OvPrP-A136)3533^+/−^ mice respectively, reflected preferential conversion by SSBP/1 prions of OvPrP^C^-V136, rapidly producing a relatively unstable OvPrP^Sc^-V136(U) conformation that was diffusely deposited in the CNS, compared to the slower conversion of OvPrP^C^-A136 to the more stable OvPrP^Sc^-A136(S) conformer which accumulated in the CNS with a punctate pattern (Figs. 1–3). Our results are consistent with the selection by the A/V136 dimorphism of SSBP/1-A136(S) and SSBP/1-V136(U) prions in Tg(OvPrP-A136)3533^+/−^ and Tg(OvPrP-V136)4166^+/−^ mice respectively. We also show that PMCA recapitulates the influence of the A/V136 polymorphism on the kinetics of SSBP/1 propagation observed in Tg mice. The general conclusions from these studies agree with previously published assessments of the mechanism of conformational selection by distinct PrP primary structures [39], [40].

Based on the rapid SSBP/1 incubation times in Tg(OvPrP-A/V136) mice, and shared conformational and distribution properties of OvPrP^Sc^ produced under these conditions with OvPrP^Sc^-V136(U) in Tg(OvPrP-V136)4166^+/−^ mice, we speculated that OvPrP-A136 played no part during the propagation of SSBP/1 prions in Tg(OvPrP-A/V) mice. To address this we used mAb PRC5 to exclusively monitor conversion of OvPrP^C^-A136. Surprisingly, in contrast to its relatively slow conversion when OvPrP^C^-A136 is expressed in isolation, co-expression with OvPrP^C^-V136 in Tg(OvPrP-A/V136) mice facilitated rapid conversion of OvPrP^C^-A136 to OvPrP^Sc^-A136. The conformation and diffuse CNS distribution of the resulting OvPrP^Sc^-A136(U) were equivalent to that of OvPrP^Sc^-V136(U) and not OvPrP^Sc^-A136(S). Collectively, these results lead us to conclude that once OvPrP^Sc^-V136(U) is formed by conversion of OvPrP^C^-V136 by SSBP/1 prions, the resulting unstable conformation induces rapid conversion of OvPrP^C^-A136 to OvPrP^Sc^-A136(U). That this outcome is dependent on allele co-expression within the host is demonstrated by the inability of the OvPrP^Sc^-V136(U) conformer to template OvPrP^C^-A136 when it is expressed in isolation.

Effects of OvPrP genotype on the propagation of scrapie prions were not controlled during the isolation and propagation of SSBP/1. Passage of SSBP/1 through Tg mice therefore allowed us to generate prions composed solely of OvPrP^Sc^-A136, OvPrP^Sc^-V136, or mixtures of both, and to draw additional conclusions about the effects of the A/V136 dimorphism of prion propagation using PMCA. Similar to our observations in Tg mice, SSBP/1 failed to convert OvPrP^C^-A136 to OvPrP^Sc^-A136 by PMCA, except in the presence of OvPrP^C^-V136 (Figs. 4 and 5). These conversion properties are shared with SSBP/1-V136(U) prions, but are distinct from SSBP/1-A136(S) prions, which show facile conversion of both OvPrP^C^-V136 and OvPrP^C^-A136. These results suggest that the SSBP/1-V136(U) is the dominant strain in the natural SSBP/1 isolate. Multiple parameters could account for this, including, but not restricted to, the effects of OvPrP genotype, for example as a result of exclusive propagation in sheep of the V136/V136 genotype, route of transmission in the infected sheep, and differential/selective prion replication in the lymphoreticular or central nervous systems of sheep. While our analyses indicate the presence of both OvPrP-V136 and OvPrP-A136 alleles in SSBB/1 (Table 1), it is important to note that SSBP/1 was derived from a pool of sheep brains of undefined genotypes. PCR approach precludes assessment of the extent to which alleles are present in a sample, raising the possibility that the one or other allele exists as a minor component in SSBP/1.

Our findings also suggest that PrP^Sc^ conformers may cross-inhibit PrP conversion. In case of SSBP/1-A136(S) prions, the presence of OvPrP^C^-V136 inhibited PMCA of OvPrP^C^-A136 (Fig. 5C). Also, while SSBP/1 seeding of PMCA reactions containing mixtures of OvPrP^C^-A136 and OvPrP^C^-V136 resulted in robust, reproducible conversion to OvPrP^Sc^-A136 as early as round one (Fig. 4H), total PrP^Sc^ production was ephemeral with subsequent PrP^Sc^ formation diminishing during rounds two to five, and conversion ultimately becoming undetectable after round six. Since early PrP^Sc^ conversion was sustained out to round 10 when OvPrP^Sc^-A136 was not produced (Fig. 4C and G), these results are consistent with inhibited conversion of OvPrP^C^-V136 to OvPrP^Sc^-V136 by OvPrP^Sc^-A136. While early (round one) PMCA conversion of PrP^Sc^ by SSBP/1 with either OvPrP^C^-V136 or mixtures of OvPrP^C^-V136 and OvPrP^C^-A136 correlates with early onset of disease following SSBP/1 infection of both Tg(OvPrP-V136) and Tg(OvPrP-A/V136) mice, the subsequent inhibitory effects of OvPrP^Sc^-A136 observed in PMCA would be impossible to detect in vivo, since Tg(OvPrP-A/V) mice succumb to the lethal effects of early PrP^Sc^ accumulation. Consistent with an inhibitory effect of OvPrP^Sc^-A136(U), prions from Tg(OvPrP-A/V) mice, while they converted OvPrP^C^-V136 in isolation, failed to convert OvPrP^C^-A136 to PrP^Sc^ in the presence of OvPrP^C^-V136 (Fig. 5D). Thus, the properties of prions from this defined genetic background differ from SSBP/1. We emphasize that, despite PCR data supporting the presence of OvPrP-A136 alleles in this isolate, SSBP/1 was derived from sheep of undefined OvPrP genotypes, rather than sheep with a defined heterozygous OvPrP-A/V136 genotype.

### Wider implications for the mechanism of prion propagation

The inter-related effects of PrP primary and higher order structures on prion transmission were addressed in the Conformational Selection Model, which proposed that strains are composed of a range of PrP^Sc^ conformers, or quasi-species, and that only a subset of PrP^Sc^ conformations is compatible with each PrP primary structure [41]. While this model also took into account the effects of polymorphic variation on prion propagation, it did so only in the context of Tg mice expressing individual PrP allele products. Transgenetic studies of the human codon 129 methionine (M)/valine (V) polymorphism, and the analogous codon 132 M/leucine (L) polymorphism in elk, indicated that these dimorphisms acted to restrict or promote the propagation of particular prion strains [39], [40]. While the responses of Tg(OvPrP-A136)3533^+/−^ and Tg(OvPrP-V136)4166^+/−^ mice are consistent with this notion, that is selection of the U conformer by OvPrP-V136, and the S conformer by OvPrP-A136, our unprecedented ability to analyze allele specific conversion in infected Tg(OvPrP-A/A136) mice reveals a more complex mechanism where mixtures of PrP variants may assist or inhibit the propagation of strains under various conditions. For example, SSBP/1 or SSBP/1-V136(U) prions facilitate conversion of OvPrP^C^-A136 to OvPrP^Sc^-A136(U) *only* in the presence of OvPrP^C^-V136. Expressed in isolation, conversion of OvPrP^C^-A136 is favored by the OvPrP^Sc^(S) conformer. Our results demonstrate that co-expression of different polymorphic forms of PrP, which would be the norm in humans and animals, have profound effects on conformational selection of prion strains.

The results reported here address the molecular mechanisms associated with the phenomenon of prion strain over-dominance first observed by Dickinson and Outram [42], and subsequently reported in other settings involving co-expression of long and short incubation time PrP alleles [43]. While this phenomenon was reconciled at the time by the assumption that TSEs were caused by unidentified viral agents, our results now indicate that the suggestion raised by those studies, namely that over-dominance most likely resulted from physical interaction of allele products of the scrapie incubation time locus during infection, was prescient. Our results support a molecular mechanism involving cross templating of an otherwise resistant allele product by a dominant prion conformer, in this case OvPrP^Sc^(U), which, we speculate, involves physical association of otherwise “susceptible” and “resistant” allele products. Consistent with the observations reported here, prion strain interference may also utilize similar mechanisms of conformational selection in a host expressing different PrP allele products infected with long and short incubation period strains with different PrP^Sc^ conformational stabilities [44], [45].

In conclusion, we have used a combination of transgenic, immunologic, and in vitro approaches to explore the mechanism by which PrP primary structure variations and the conformations enciphered by different prion strains interact to control TSE propagation. While our results support previous studies indicating that PrP susceptibility polymorphisms, expressed in isolation, act to restrict or promote the propagation of particular prion conformers, we now show that under conditions of allele co-expression a dominant conformer may alter the conversion potential of an otherwise resistant PrP polymorphic variant to an unfavorable prion strain. While such responses are analogous to the phenotypic expression of genetically determined heritable traits, dominant prion conformers act epigenetically by means of protein-mediated conformational templating. By expanding the range of possible conformations adoptable by a particular prion protein primary structure, such interactive effects provide a mechanism for promoting strain fitness, and, we speculate, strain diversification. While the precise number scrapie strains in sheep and goats remains uncertain, the description of at least 24 additional major sheep *PRNP* polymorphisms, and combinations thereof, is likely to have a significant influence on strain diversity.

## Materials and Methods

### Ethics statement

All animal work was conducted according to the National Institutes of Health guidelines for housing and care of laboratory animals, and performed under protocols approved by the Colorado State University Institutional Animal Care and Use Committee, with approval number 11-2996A.

### Transgenic mice

Sequences upstream of codon 44 of the OvPrP-A136 and V136 coding sequences were replaced with the corresponding sequence from mouse PrP. The resulting constructs contained the OvPrP coding sequence, except for addition of an extra residue for glycine at codon 31, and the mouse PrP N-terminal signal peptide instead of OvPrP signal peptide. Tg mice were generated by cloning the OvPrP-A136 and OvPrP-V136 expression constructs into the cosSHa.Tet cosmid vector [34], and microinjection of embryos from inbred *Prnp* ^0/0^/FVB mice. Tg founders were identified by PCR screening of genomic DNA isolated from tail snips. Founder mice were mated with inbred *Prnp* ^0/0^/FVB mice, and generally maintained with the transgene in the hemizygous state, with Tg mice identified by PCR screening of genomic DNA from weanlings. It was also possible to generate homozygous counterparts of each line, and Tg(OvPrP-A/V136) mice were generated by crossing homozygous Tg(OvPrP-V136)4166^+/+^ mice with homozygous Tg(OvPrP-A136)3533^+/+^ mice. We used immuno-dot blotting and Western blotting with mAb 6H4 (Prionics, Schlieren, Switzerland) to estimate the levels of OvPrP expression. Tg mice subsequently shipped to and maintained in Edinburgh were crossed onto the *Prnp* ^0/0^/129Ola background [46].

### Inocula

SSBP/1 originated as a homogenate of three natural scrapie brains that were subsequently passaged mostly through Cheviot sheep at the Neuropathogenesis Unit (NPU), Edinburgh UK [15], [16]. CH1641 is a naturally infected cheviot sheep from the NPU flock [17]. The presence of OvPrP-A136 or OvPrP-V136 alleles in these samples was ascertained by restriction fragment length polymorphism analysis of the PCR amplified *PRNP* coding sequences.

Ten % mouse brain homogenates (w/v) were prepared in phosphate-buffered saline (PBS) lacking calcium and magnesium ions by repeated extrusion through 18- and 21-gauge needles. Sheep brain homogenates (10%) in PBS were prepared by repeated extrusion through 14-gauge, followed by 18- to 28-gauge needles in PBS. Total protein content was determined by bicinchonic acid (BCA) assay (Pierce Biotechnology, Inc.).

### Determination of incubation times

Anesthetized mice were inoculated intracerebrally with 30 µl of 1% (w/v) brain extracts prepared and diluted in PBS.

General health was monitored daily. Onset of prion disease was determined by observation of the progressive development of at least three of the following clinical signs: truncal ataxia, loss of extensor reflex, difficulty righting from a supine position, plastic tail, head bobbing or tilting, kyphotic posture, circling and paresis/paralysis. Animals were diagnosed when at least two investigators agreed with the manifestation of these signs. Incubation time is defined as the period between the time of inoculation to the day on which subsequently progressive clinical signs were initially recorded.

### Analyses of PCR amplified *PRNP* coding sequences

Brain homogenates containing 500 µg protein were digested with 400 µg/ml proteinase K (PK) in 0.4 M NaCl, 10 mM Tris–HCl, pH 8.0, 2 mM EDTA, pH 8.0, and 2% SDS at 55°C overnight. Genomic DNA was precipitated with isopropanol. The partial OvPrP coding sequence was amplified by PCR with the forward and reverse primers: 5′-GGACAGGGCAGTCCTGGA-3′, 5′-GTGATGCACATTTGCTCCACCACT-3′. PCR products were purified with QIAquick Gel Extraction kit (QIAGEN Science, MA, USA), digested with BspH I that only recognizes the OvPrP-V136 allele, and the products were resolved on a 1.2% agarose gel.

### Protein misfolding cyclic amplification

Tg mice were perfused with PBS/5 mM EDTA. Ten % brain homogenates (w/v) were prepared in PBS containing 150 mM NaCl, 1.0% Triton X-100, and the complete TM cocktail of protease inhibitors (Roche, Mannheim, Germany). Samples were clarified by brief, low-speed centrifugation. Protein concentrations of brain homogenates used as substrates for PMCA were adjusted to contain equivalent amounts of OvPrP-A136 or OvPrP-V136, based on the estimated relative levels of transgene expression. Substrates in which OvPrP-A136 and OvPrP-V136 were mixed were adjusted based on the estimated relative levels of transgene expression, so that approximately equal amounts of each allele product were present in the PMCA reaction. PMCA reactions were performed as described previously [20], [47] at a seed to substrate ratio of 1∶180. One cycle corresponded to 20 seconds of sonication followed by 30 minutes incubation at 37°C. Controls samples were incubated for the same duration at 37°C without sonication. Amplified and control samples were digested with PK at a final concentration of 0.33 µg/µl and analyzed on western blots using mAbs 6H4 or PRC5.

### Western blotting

Brain homogenates and cell lysates were digested with 100 µg/ml or 30 µg/ml of PK respectively (Roche, Mannheim, Germany) in cold lysis buffer for 1 h at 37°C. Digestion was terminated with phenylmethylsulfonyl fluoride at a final concentration of 2 µM. Samples were boiled for 10 min in the absence of β-meracaptoethanol [14] and proteins were resolved by SDS-PAGE and transferred to polyvinylidenedifluoride Immobilon (PVDF)-FL membranes (Millipore, Billerica, USA). Membranes were probed with primary mAbs followed by horseradish peroxidase–conjugated anti-mouse secondary antibody (GE Healthcare, Little Chalfont, UK). Protein was visualized by chemiluminescence using ECL Plus (GE Healthcare, Piscataway, USA) and an FLA-5000 scanner (Fujifilm Life Science, Woodbridge, USA).

### Conformational stability assay

Brain homogenates containing 5 µg protein were incubated with various concentrations of guanidine hydrochloride (GdnHCl) in 96-well plates for 1 h at room temperature. Samples were adjusted with PBS to a final of concentration of GdnHCl of 0.5 M and transferred onto nitrocellulose (Whatman GmbH, Dassel, Germany) using a dot blot apparatus. After two PBS washes, the membrane was air-dried for 1 h, then incubated with 5 µg/mL PK in 50 mM Tris-HCl, pH 8.0, 150 mM NaCl, 0.5% sodium deoxycholate, 0.5% Igepal CA-630 for 1 h at 37°C. PK was inactivated with 2 mM PMSF. The membrane was incubated in 3 M guanidine thiocyanate in Tris-HCl, pH 7.8 for 10 min at room temperature. After four washes with PBS, the membrane was blocked with 5% nonfat milk in TBST for 1 h, and probed with mAbs 6H4 (1∶20,000) or PRC5 (1∶5000) overnight at 4°C, followed by HRP-conjugated goat anti-mouse IgG secondary antibody. The membrane was developed with ECL Plus and scanned with GE image quant 4000. The signal was analyzed with ImageQuant TL 7.0 software.

### Histoblotting

Histoblots were produced and analyzed according to previously described protocols [23]. Images were captured with a NikonDMX 1200F digital camera in conjunction with Metamorph software (Molecular Devices).
